# Pharmacist-Mediated Deprescribing in Long-Term Care Facilities: A Systematic Review

**DOI:** 10.3390/pharmacy13010003

**Published:** 2025-01-04

**Authors:** João Rafael Gonçalves, Neuza Magalhães, Sara Machado, Isabel Ramalhinho, Afonso Miguel Cavaco

**Affiliations:** 1 iMed.ULisboa—Research Institute for Medicines, Faculty of Pharmacy, University of Lisbon, 1649-003 Lisboa, Portugal; s.machado@campus.ul.pt (S.M.);; 2 Faculty of Science and Technology, University of Algarve, Campus de Gambelas, 8005-139 Faro, Portugal; nea.maga@gmail.com (N.M.); iramalhinho@ualg.pt (I.R.)

**Keywords:** long-term care, deprescribing, pharmacist, long-term care facilities, nursing homes, care homes

## Abstract

Multimorbidity and polypharmacy are prevalent among Long-Term Care (LTC) users. Older people, who most use LTC services, are more prone to drug-related problems, which polypharmacy aggravates. Deprescribing is a key intervention to address polypharmacy and inappropriate medication. Evidence shows that pharmacists’ expertise in medicines and their growing involvement in clinical-oriented activities have proven to play an essential role across many healthcare settings, including LTC. Thus, this study aimed to identify and assess LTC pharmacist-mediated deprescribing. A systematic review was undertaken following the PRISMA checklist, using three literature databases (PubMed, Scopus, and Web of Knowledge). A set of 18 keywords, divided into three domains (professional, type of care, and type of setting), were combined into search equations. The studies selected were assessed through the Quality Assessment Tool for Quantitative Studies. Fifteen studies met the inclusion criteria out of 288 initial hits. Pharmacist-mediated deprescribing was divided into specific (targeted to a medicine group) and non-specific. Half of the studies were graded as low quality (53%). In total, the studies enrolled 6928 patients and 45 pharmacists. The ATC groups A, C, M, and N, as well as medicines with anticholinergic properties, were the most addressed medicines groups. Acceptance rates of pharmacists’ recommendations ranged between 30% and 100%. Generically, the number of medicines was reduced after the intervention. Mixed results were found for falls and quality of life outcomes. Cost savings associated with the interventions ranged from neutral to as high as 3800 €/patient/year. Barriers to deprescribing were mainly linked to patients’ or family members’ refusal to change. In conclusion, pharmacist-mediated deprescribing seems feasible in LTC. The studies’ methodological heterogeneity hampers robust comparisons and conclusions. The medicine groups targeted by deprescribing can help tailor interventions to optimize the use of medicines in LTC. A detailed understanding of barriers and enablers to deprescribing would support developing and implementing these interventions.

## 1. Introduction

Over the last decades, the World Health Organization (WHO) has urged countries to develop and improve Long-Term Care (LTC) systems, mainly because of the marked global aging [1].

Long-Term Care is defined as the combination of health, social, and personal care aimed at people who, permanently or temporarily and regardless of age, experience reduced functionality due to aging, chronic illness, or after an emergency episode [1,2]. LTC can be delivered in domiciliary or institutional settings (ambulatory or inpatient care) [1]. Regarding inpatient settings, many terms are employed, such as skilled-nursing facilities, care homes, and Long-Term Care facilities (LTCFs) [3,4]. Often, the designation differs according to the preponderance of health or social care delivered in these facilities. As stated by the Organisation for Economic Co-operation and Development, “social LTC” is offered in “assisted living arrangements and other kinds of protected housing for persons with functional limitations […]” and includes services such as “housekeeping, day-care social services […]; and transport to and from day-care facilities” [5]. On the other hand, “health LTC” consists of “a range of medical and personal care services that are consumed with the primary goal of alleviating pain and suffering and reducing or managing the deterioration in health status in patients with a degree of long-term dependency or similar social services for persons with functional limitations” [5].

The prevalence of multimorbidity (co-occurrence of two or more chronic conditions) [6] and polypharmacy (concurrent use of 5 or more medicines) is high among LTC recipients [7]. Evidence suggests a prevalence of polypharmacy in LTCFs between 75% and 91% [8,9]. Considering the age-related pharmacokinetics and pharmacodynamics changes [10] and the under-representation of geriatric populations in clinical trials [11], this prevalence represents a significant concern, given the association between inappropriate polypharmacy and negative health-related outcomes in older people, e.g., adverse drug events, hospitalization, mortality [12,13]. Inappropriate polypharmacy can be defined as “where the potential harms of individual medicines, prescribed in combination with several other medicines, outweigh the benefits” [14].

Many strategies have been developed to optimize the use of medicines in LTC contexts, e.g., educational/training activities, development and employment of implicit or explicit criteria to identify Potentially Inappropriate Medication (PIM) or underprescribing [15,16,17]. One of the strategies to tackle inappropriate polypharmacy is the process of deprescribing. The WHO defines deprescribing as “tapering, stopping, discontinuing, or withdrawing medicines, with the goal of managing polypharmacy and improving outcomes” [18]. A relevant medication-related problem associated with deprescribing practices is the onset of Adverse Drug Withdrawal Events (ADWE), defined as a clinically significant set of symptoms or signs caused by the removal of a drug [19].

Pharmacists are commonly identified as medication experts, and the literature suggests a positive link between pharmacists’ interventions in optimizing pharmacotherapy in LTC settings and medicine-related outcomes [15,16,17,20].

Considering the above, this study aimed to map pharmacist-mediated deprescribing interventions in LTC settings predominantly delivering “Health LTC”, to assess the quality of the studies, and to characterize the nature of the interventions and the most significant medicines and medicines groups addressed.

## 2. Materials and Methods

This study is a systematic review guided by the Preferred Reporting Items for Systematic Reviews and Meta-Analyses (PRISMA) statement standards [21]. Three researchers independently searched the literature during the last week of September 2024.

### 2.1. Data Sources and Search Strategy

Searching was conducted in three online databases from inception: PubMed, Scopus, and Web of Science. Eighteen keywords referring to the type of intervention, type of care, and study professional were combined into the following search equation, applied to all fields: (“deprescribing” OR “medication withdrawal” OR “medication discontinuation” OR “deprescription*” OR “stop* medic*” OR “unnecessary treatment” OR “medication cessation” OR “rationalization”) AND (“Long-term care” OR “long-term care facility” OR “skilled nursing facility” OR “care home” OR “nursing home” OR “continuing care” OR “sub-acute care”) AND (“Pharmacist*” OR “Pharmaceutical service*” OR “Pharmaceutical care”). All team members agreed on the final set of keywords and search equations. The studies were carefully screened through a two-step method: firstly, by assessing the article title and abstract, and secondly, by a full-text analysis. Broadly, this review sought to identify studies linking pharmacists’ interventions to deprescribing outcomes. Inclusion and exclusion criteria are detailed in Table 1. The included studies were appraised (described below) and qualitatively synthesized.

### 2.2. Data Extraction and Quality Assessment

The studies’ quality assessment was performed using the Quality Assessment Tool for Quantitative Studies (QATQS), developed by the Effective Public Health Practice Project [22]. The evaluation was based on five components: selection bias, study design, confounders, data collection, and dropouts. The sixth component, blinding, was excluded once its rating was generally impossible to deliver, given the nature of most studies. Each of the five components was rated weak, moderate, or strong. After discussion and agreement between reviewers, global ratings were achieved using the following rule, as defined by the QATQS: strong, absence of weak ratings; moderate, one weak rating; weak, two or more weak ratings.

## 3. Results

**Figure 1:**
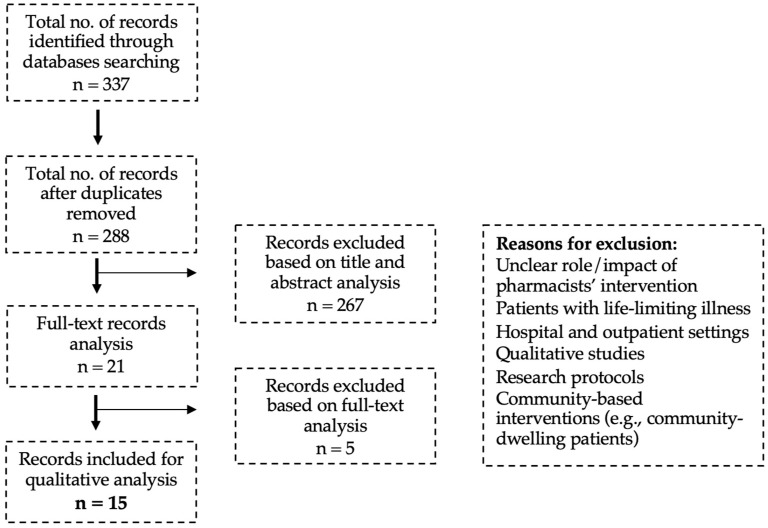
PRISMA flow diagram for the study selection process.

The main results of the screening process are presented in Figure 1. From the 288 hits obtained and after duplicate removal, 15 was the final number of studies agreed upon for full-textual analysis and qualitative synthesis.

### 3.1. Summary of Studies

Table 2 summarizes the main findings from each selected study presented according to the authors and year of publication, country, study design, number of patients and their sociodemographic characteristics, number of pharmacists and LTCFs involved, and the most significant result. Most studies were developed in Canada (n = 3), the United Kingdom (n = 3), Singapore (n = 2), and the United States of America (n = 2). The remaining studies (n = 5) were developed in the Netherlands, Switzerland, New Zealand, Spain, and Australia. In total, 6928 patients and 45 pharmacists were enrolled.

### 3.2. Quality Assessment

Regarding quality assessment, the 15 selected studies were quality-graded using the QATQS tool (Table 3). Approximately 50% of studies were classified as weak (n = 8) and 30% as strong (n = 5). “Selection bias” and “confounders” contributed the most to these results.

### 3.3. Mapping of Deprescribing Interventions

Considering the studies’ heterogeneity and as a manner to systematize the evidence better, deprescribing interventions were categorized as specific and non-specific, i.e., targeted to particular medicine groups or not. The medicines were classified according to the Anatomical Therapeutic Chemical (ATC) classification system [38].

The outline features of interventions varied across studies. For most studies, medication review was the initial step, with studies also reporting the participation of pharmacists in interviews to construct the clinical history [23,26,31,35,36]. Nurses and physicians were the most involved healthcare professionals (particularly physicians) and psychologists [36]. When mentioned or applicable, recommendations were delivered and discussed with the physician face-to-face [23,26,27,31,37], through notes written in the medical record [32,34,35,36], communicated through a nurse [33], or employing a combination of direct (in person) and indirect forms of communication [24,28]. In other studies, given the expanded role of pharmacists in prescribing, detection of inappropriate medication and changes to the pharmacotherapeutic plan were actioned by pharmacists [24,27]. Potential medicines to deprescribe were discussed with patients or legal representatives in some studies [23,26,27,29,30,31].

#### 3.3.1. Specific Deprescribing (n = 7)

Seven studies evaluated pharmacist-mediated deprescribing interventions on specific medicines or medicines groups, mainly belonging to the ATC groups alimentary tract and metabolism (A) and nervous system (N). In total, 619 patients and 10 pharmacists were involved in specific deprescribing interventions.

Three studies addressed medicines with sedatives, hypnotic or anticholinergic properties [23,28,32]. One study [32] targeted specific medicines—trazodone, diphenhydramine, hydroxyzine, temazepam, flurazepam, diazepam, alprazolam, lorazepam, and zolpidem—according to the recommendations of the American Geriatrics Society Beers criteria [39] and the Centers for Medicare & Medicaid Services Guidance’s Tag F329 [40]. The two other studies employed different assessment tools, namely the Drug Burden Index [41] and the Psychotropic Drug Safety Initiative [42]. Most medicines the three studies address are classified in the ATC group nervous system (N).

The studies by Lee et al. [34] and Tandun et al. [37] targeted the deprescribing of proton pump inhibitors (PPI). In both studies, pantoprazole (A02BC02) and esomeprazole (A02BC05) were the PPI involved in deprescribing.

Ee et al. [31] and Sanz-Tamargo et al. [36] described interventions towards a set of medicine groups employed to manage several diseases and symptoms. The ATC groups addressed were A (i.e., PPIs, A02BC; laxatives, A06A; antiemetics, A04A; calcium, A12AA), B (i.e., iron, B03A; acetylsalicylic acid, B01AC06), C (i.e., furosemide, C03CA01; statins, C10AA); G (i.e., drugs for urinary frequency and incontinence, G04BD); M (i.e., antiinflammatory and antirheumatic products, non-steroids, M01A), and N (i.e., paracetamol, N02BE01; antidepressants, N06A; benzodiazepines, N05BA; anticholinesterases, N06D; memantine, N06DX01; antipsychotics, N05A).

Evaluation of ADWEs was reported in five studies [23,31,34,36,37]. Reduction or discontinuation of target medicines leads to the development of signs and symptoms, such as diarrhea and insomnia as a result of codeine and amitriptyline withdrawal, respectively [23]; recurrence of pain and reinitiation of analgesics [31]; pyrosis due to PPI withdrawal [34,36,37]; and worsening in neuropsychiatric symptoms caused by antipsychotic withdrawal and insomnia/anxiety caused by benzodiazepines withdrawal [36].

#### 3.3.2. Non-Specific Deprescribing (n = 8)

Eight studies evaluated pharmacist-mediated deprescribing interventions not targeted to specific medicines or medicine classes [24,25,26,27,29,30,33,35]. In total, 35 pharmacists and 6309 patients were involved in non-specific deprescribing interventions.

Identification of drug-related problems and consequent suggestion for deprescribing was supported by different tools, i.e., Screening Tool of Older Persons’ Prescriptions [26,27,30,33,43]; AGS Beers criteria [26,35,39]; Good Palliative-Good Practice tool [35,44]; Optimising Safe and Appropriate Medicines Use [25,45]; Systematic Tool to Reduce Inappropriate Prescribing [29,46]; Drug Burden Index (DBI) [35,41]; Anticholinergic Risk Scale (ARS) [35,47].

Lack of ongoing indication was the main reason for deprescribing, followed by high dosage [26] and other safety reasons (e.g., duplication) [24,27,30].

The most frequent medicines addressed belong to the following ATC groups: A (e.g., PPIs, A02BC; drugs for constipation, A06A; vitamins, A11) [25,26,27,29,33,35], C (e.g., lipid modifying agents, C10; antihypertensives, C02) [25,26,27,29,33], N (e.g., antipsychotics, N05A, anxiolytics, N05B, antidepressants, N06A) [25,26,29,35], and M (e.g., antiinflammatory and antirheumatic products, non-steroids, M01A; drugs affecting bone structure and mineralization, M05B) [27,30]. Kua et al. [33] and Cateau et al. [30] studies have identified anticholinergic medicines as a preponderant group in deprescribing recommendations.

ADWE were detailed in four studies [26,27,29,30], e.g., gastroesophageal reflux disease symptoms returned after PPI withdrawal; hallucination and anxiety after quetiapine withdrawal and risperidone taper, respectively; increased blood pressure and leg edema after diuretics deprescribing (CO3A, C03CA); gout flare after febuxostat dose reduction.

### 3.4. Outcomes of Deprescribing

Considering the set of 15 studies (i.e., specific and non-specific deprescribing), acceptance rates of pharmacists’ recommendations ranged between [25–50%] [25,30,32], [51–74%] [28], and [75–100%] [23,26,29,33,34,35,36]. Five studies estimated financial savings, varying as follows: no cost savings [31], 80 € [36], 90 € [24], 250 € [27], and 3800 € [33] (patient/year). Some studies determined the impact of deprescribing interventions at the end of the follow-up periods (from 2 months to 12 months), presenting different results [23,26,30,31,33,35,37]: Balsom et al. and Kua et al. reported a decrease in medicines per patient, while Cateau et al. reached no statistically significant impact. Ailabouni et al. detected a reduction in fall rate, inversely to Cateau et al. and Kua et al. Quality of life (QoL) was assessed in two studies, with Ailabouni et al. reporting no changes at six months and Cateau et al. suggesting a reduction in QoL. The need for re-prescribing was necessary, i.e., in one study, 50% of patients were re-prescribed, at least, one medicine [23]; although for remaining studies, the need for re-prescribing ranged between [7.5–30%] [29,33,34,35,36,37].

Seven studies were assessed as of moderate and strong quality. The interventions described belonged to specific (n = 3) and non-specific deprescribing, i.e., both sets of specific and non-specific deprescribing are composed of 50% of high-quality studies. Medicines with anticholinergic and sedative properties and belonging to the ATC groups alimentary tract and metabolism (e.g., proton pump inhibitors, antiemetics, laxatives), cardiovascular system, and nervous system (e.g., antidepressants, antipsychotics, paracetamol) were commonly addressed throughout studies. Acceptance rates of pharmacists by physicians ranged between 49% [30] and 92.5% [36]. The impact of pharmacists in reducing the number of medicines was reported as positive in all studies but one [31].

Barriers and facilitators of deprescribing were not detailed in all studies. Some studies state that physicians “felt not comfortable” [23], “hesitant” [32], or no “clear reason was given” [23,34] Patients or patient legal representatives also rejected changes suggested by pharmacists [26,29,32,36]. Concerns about worsening symptoms and “reluctance to discontinue medication prescribed by a specialist” were also mentioned [26]. Bell et al. referred to mixed preferences of physicians for pharmacists to communicate (face-to-face, electronic note communication, or both) [28]. Tandum et al. hypothesizes that previous contact between pharmacists and patients increases acceptance rates because pharmacists are “more familiar with residents’ conditions and symptoms”, leading to “greater confidence in recommending” deprescribing [37].

## 4. Discussion

Several reviews on deprescribing interventions have been published in the literature, highlighting the relevance to healthcare delivery, particularly towards polymedicated older populations [48,49,50,51,52]. People aged ≥ 65 years represent the majority of LTC users, and multimorbidity and polypharmacy are highly prevalent among LTC populations [8,9,53]. Consequently, medicines are a typical therapeutic resource employed in LTC settings, with pharmacists positively impacting clinical and economic outcomes [15,16,17]. In 2019, Alves et al. noted the lack of systematized evidence on “deprescribing in care home settings” [24]. The present review not only relates LTC and deprescribing but also evaluates the impact of pharmacists on this axle, filling a gap in evidence.

The quality of studies is low (53% of studies rated as weak), which aligns with other systematic reviews assessing medicine-related interventions in LTC [4,15]. Adding to the diversity of terminology and concepts associated with LTC worldwide [54], both factors may hamper comparisons. Nonetheless, outcomes assessed in studies included in this review are comparable with other pharmacist-mediated interventions in LTC summarized in recent systematic reviews [15,16,17]. The acceptance rates of pharmacists’ recommendations identified in the present review (30–100%) are similar to other reviews [49]. The evidence reported here also suggests a positive impact in reducing the number of medicines per patient and cost savings. Inversely, the effect of deprescribing on falls, hospitalization, and mortality was not evaluated in most present studies, and the evidence from other systematic reviews is heterogeneous [16,17]. For these reasons, the body of evidence could be strengthened, especially by improving study quality and establishing a standard set of indicators.

In a qualitative meta-synthesis, stakeholders perceive the role of pharmacists in deprescribing as valuable, particularly in clinical aspects of medication management [55]. Other systematic reviews, including pharmacist-led, physician-led, or multidisciplinary team-led deprescribing, reveal a positive impact or mixed results [49,50,52]. A decrease in the number of medicines and/or doses is shared between all reviews. High heterogeneity in outcomes measures and reporting was also identified in the literature [51]. For outcomes commonly measured between the present study and other reviews, the impact of deprescribing on costs, falls, and QoL seems to be positive, albeit unclear findings were found. For instance, regarding fall rates, the review by Iyer et al. refers to a reduction in falls; in contrast, the review by Thillainadesan et al. shows little impact, i.e., four studies reported the effect on falls, with only one showing a statistically significant reduction [50,52]. The same occurred in the present review, with three studies assessing fall rate [23,30,33] and only one reaching a statistically significant decrease for this outcome [23]. Pharmacist-mediated deprescribing seems as feasible and effective as physician- or multidisciplinary team-led deprescribing. However, some limitations of the present study (i.e., methodological heterogeneity, low quality, and small number of the studies included) should be considered in this appraisal.

Intervention differences indicate that legal frameworks and pharmacy education vary across countries. In some studies, the implementation of deprescribing is not “physician-dependent”, given the expanded role of pharmacists in prescribing [56]; Cateau et al. [30] mentioned that “prior to the start of the trial, the pharmacists took part in a 3-day postgraduate education session on the methodology of performing medication reviews, as this is not part of the pharmacy curriculum in Switzerland”. Both aspects may indicate that legislation and education can be crucial to developing pharmacist-mediated deprescribing.

Studies included in this review and comparable reviews mentioned above [49,50,52] share target medicines or medicine classes. The ATC groups A (e.g., A02, A06, A11), C (e.g., C02, C03, C10), M (e.g., M01 and M05), and N (e.g., N05 and N06A) and medicines with anticholinergic properties are frequently addressed. Some reasons can justify this fact, such as the employment of common criteria supporting deprescribing (i.e., START/STOP, AGS Beers criteria, DBI, ARS) and study population (i.e., older people). Particularly in LTC, evidence shows that the ATC groups mentioned above frame the most common PIMs found in research [15,57]. These common grounds can help establish fields of intervention that are helpful throughout settings and populations, considering the importance of person-centered integrated care [58].

Moreover, broader implementation of evidence-based practices into daily practice takes several years [59]. Better and faster interaction between research and knowledge users aimed to improve healthcare systems is a concern worldwide [60,61]. Thus, this evidence could work as a starting point to tailor interventions towards optimized usage of medicines in LTC, framed by Implementation Science principles that could be followed “to ensure that research investments maximize healthcare value and improve public health”, as Bauer et al. stated [62]. Additionally, barriers and enablers of deprescribing should be further explored. Several barriers were mapped in the literature related to patients/family members and healthcare professionals (e.g., fear of symptoms return and negative impact on QoL, multiple medical providers, trust in healthcare professionals, skill and knowledge lack) [63,64,65]. Some reasons are shared with those described in the present review’s studies, such as concern about worsening symptoms. On the other hand, examples of enablers identified in the literature encompass safety concerns (e.g., the patient experienced an adverse effect), deprescribing education (e.g., education on medication cessation and expectations upon discontinuation), or prescriber’s knowledge and skills on PIMs identification and management [63,65]. Pharmacists are medicine experts and commonly deliver education and training activities, including in LTC [15,17], where pharmacists’ involvement can improve the quality and extent of deprescribing.

## 5. Conclusions

Considering the prevalence of polypharmacy among LTC users, deprescribing is a vital intervention to tackle this challenge. Deprescribing interventions in LTC described in the literature target medicines associated with poorer health outcomes to older populations, known as being more prone to experience adverse drug events. Despite the overall low quality of studies and methodological heterogeneity, evidence suggests a positive impact of pharmacist-mediated deprescribing. It is possible to identify a shared set of the most addressed ATC groups across studies (A, C, M, and N). Interventions are well-tolerated by patients, caregivers, and healthcare professionals, although reasons explaining different acceptance rates should be further evaluated, along with the barriers and enablers to pharmacist-mediated deprescribing. Future research should be designed to increase the robustness of evidence by assessing the impact of deprescribing on important outcomes, such as falls, hospitalization, quality of life, and mortality.

## Figures and Tables

**Table 1:** Exclusion and inclusion criteria.

Category	Inclusion Criteria	Exclusion Criteria
*Population*	Pharmacists working in LTCFs or similar institutional LTC settings with a well-defined role within the LTC healthcare team	Deprescribing interventions initiated and managed by other healthcare professionals
*Geography*	No limit	--
*Study period*	No limit	--
*Settings*	Long-Term Care facilities or similar settings delivering “Health LTC”	Outpatient settings Home-based settings Acute hospital wards Primary care settings Palliative or hospice care
*Intervention*	Deprescribing interventions provided by pharmacists	Interventions on which pharmacists’ participation is not well defined; exclusively educational interventions
*Outcomes*	Outcomes attributable to pharmacists’ intervention, e.g., PIMs identified; acceptance rate of pharmacists’ recommendations (by healthcare professionals, patients, or caregivers); clinical outcomes (falls, hospitalization, mortality); pharmacoeconomic impact	Exclusively assessment of knowledge, awareness, and satisfaction outcomes; exclusively identification of enablers and barriers toward deprescribing
*Publication language*	English, Portuguese, Spanish, French	--
*Study design*	Experimental and observational analytical studies	Systematic reviews Qualitative studies

**Table 2:** Summary of included studies.

Author (Year)	Country	Study Design	No. of Patients Enrolled (Characteristics)	No. of Pharmacists Involved (No. of LTCFs)	Most Significant Results
Ailabouni et al. (2019) [23]	New Zealand	Cohort (before-and-after)	46 (≥65 years; female, 74%)	1 (n = 3)	Change in Drug Burden Index (DBI). A total of 72% of the deprescribing recommendations suggested were implemented. A total of 96% of the residents agreed to the recommendations. Reduction in DBI scores by 0.34, number of falls, and adverse drug events.
Alves et al. (2019) [24]	United Kingdom	Cross-sectional	5035 (>60 years)	25 (n = 499)	In 2018–2019: 1264 deprescribing interventions with a potential yearly saving of approx. 129,000 €. Total savings: 500,000 € over 5 years.
Andreassen et al. (2016) [25]	United Kingdom	Cross-sectional (sub-analysis of a randomized-controlled trial)	106 (median 86 years [56–98]; female, 66%)	2 (n = 30)	A total of 346 PIMs were identified. A total of 90% of residents with Type 2 diabetes had at least one Potentially Inappropriate Medication. The physician unreservedly endorsed 39% of the suggested recommendations.
Balsom et al. (2020) [26]	Canada	Randomized controlled trial	45 (intervention, n = 22; median 84.3 years; female, 45.5%|control, n = 23; median 84.5 years; female, 56.5%)	1 (n = 1)	A total of 78 recommendations were made; 67 (85.9%) were accepted, and 57 (85.1%) were successfully implemented. The average number of medications taken by residents in the intervention group was 2.68 less than the control group (*p* < 0.02; 95% CI −4.284, −1.071) at 3 months and 2.88 less (*p* = 0.02, 95% CI −4.543, −1.112) at 6 months.
Baquir et al. (2017) [27]	United Kingdom	Cohort (before-and-after)	422 (median 85.5 years; female, 77.7%)	Not mentioned (n = 20)	A total of 70% of patients had at least one medicine stopped; 52.3% of medicines were stopped; mean number of medicines stopped: 2.36 ± 1.53. There is no difference between the number of medicines stopped by pharmacists and physicians (*p* = 0.9702; 95% CI −0.39 to 0.38).
Bell et al. (2020) [28]	United States of America	Cohort (before-and-after)	21 (median 83 years [69–95], male, 100%)	1 (n = 1)	Potentially inappropriate psychotropic medication use. Recommendations were accepted 66% of the time.
Blenke et al. (2018) [29]	The Netherlands	Cohort (before-and-after)	45 (mean 82.8 ± 7.0 years; male, 26.7%)	4 (n = 3)	Approval rate of 94.3%. 90-day implementation rate 84.8%. A total of 55.3% of the recommended changes to deprescribe concerned 10 drug groups.
Cateau et al. (2021) [30]	Switzerland	Controlled clinical trial	58 (intervention, n = 31; median 87 [80–91] years; female, 52%|control, n = 27; median 84 [78–88] years; female, 74%)	Not mentioned (n = 7)	The pharmacists proposed 169 modifications, and 49% were accepted and implemented. Defined Daily Doses (DDD) significantly reduced in the intervention group (IRR 0.763, 95% CI [0.594, 0.979]), with a more marked effect at chronic drugs, with a 28% reduction (IRR 0.716, 95% CI [0.546, 0.938]) in the number of long-term PIM DDDs.
Ee et al. (2018) [31]	Singapore	Randomized controlled trial	190 (intervention, n = 94; median 72.8 ± 10.7 years; female, 45.7%|control, n = 96; 68.6 ± 10.0 years; female, 64.5%)	3 (n = 1)	The deprescribing of symptomatic control medications did not lead to increased cost savings. The reduction in the total number of medications was higher in the control group, but the difference between the groups was insignificant.
Gemelli et al. (2016) [32]	United States of America	Cohort (before-and-after)	36 (male, n = 11, 76.6 ± 8.1 years; female, n = 25, 82.64 ± 6.9 years)	Not mentioned (n = 11)	Gradual dose reductions/discontinuation of select sedatives/hypnotics were accepted for 48.7% of residents.
Kua et al. (2020) [33]	Singapore	Randomized controlled trial	295 (intervention, n = 142; median 80.02 ± 9.58 years; female, 52.8%|control, n = 153; median 80.57 ± 9.42 years; female, 58.17%)	Not mentioned (n=4)	The physician accepted 75% of the recommendations. The intervention was associated with a reduction in mortality [HR 0.16, 95% CI 0.07, 0.41; *p* < 0.001], number of hospitalizations (HR 0.16, 95% CI 0.10, 0.26; *p* < 0.001) and medicine usage. Cost saving of 10 €/patient.
Lee et al. (2017) [34]	Canada	Cohort (before-and-after)	27 (median 80 ± 11.8 years; female, 64%)	3 (n = 1)	The recommendations to discontinue therapy were accepted for 27 (96%) patients. At 8 weeks after the intervention, 70% of these residents were still asymptomatic and did not require reinitiation of medications.
Quek et al. (2023) [35]	Australia	Randomized controlled trial	303 (102 in the blinded intervention group, 101 in the open intervention, and 100 in the blinded control group (≥65 years old))	3 (n = 17)	A total of 77% (941/1222) of deprescribing recommendations were accepted. Of the accepted recommendations, 74% (692/941) were successfully implemented at the end of the follow-up visit at 12 months. The most common reason for deprescribing was because medications were no longer needed (42%, 513/1231).
Sanz-Tamargo et al. (2019) [36]	Spain	Cohort analytic (two groups before-and-after)	241 (intervention, n = 119; median 81.5 [65–95] years; female, 68.8%|control, n = 122; median 81 [65–96] years; female, 67.2%)	Not mentioned (n = 2)	Acceptance rate of 98.9%. A total of 175 PIMs were detected, and 92.5% were deprescribed (1.4 per patient) Annual saving of 9525.25 €.
Tandun et al. (2019) [37]	Canada	Cohort (before-and-after)	58 (median 80 ± 12.1 years; female, 76%)	2 (n = 2)	A total of 62.5% of patients were recommended to have proton pump inhibitors deprescribed. Four months after intervention, recommendations were kept for 80% of patients.

**Table 3:** Quality assessment of included studies.

Author (Year)	Selection Bias	Study Design	Confounders	Data Collection	Withdrawals and Dropouts	Final Rating
Ailabouni (2019) [23]	moderate	moderate	weak	moderate	strong	**Moderate**
Alves (2019) [24]	weak	weak	weak	moderate	not app.	**Weak**
Andreassen (2016) [25]	moderate	weak	weak	strong	not app.	**Weak**
Balsom (2020) [26]	moderate	strong	strong	strong	strong	**Strong**
Baqir (2017) [27]	weak	moderate	weak	moderate	not app.	**Weak**
Bell (2020) [28]	weak	moderate	weak	moderate	weak	**Weak**
Blenke (2018) [29]	weak	moderate	weak	strong	moderate	**Weak**
Cateau (2021) [30]	weak	strong	moderate	strong	moderate	**Moderate**
Ee (2018) [31]	moderate	strong	moderate	strong	strong	**Strong**
Gemelli (2016) [32]	weak	moderate	weak	moderate	not app.	**Weak**
Kua (2020) [33]	moderate	strong	moderate	strong	strong	**Strong**
Lee (2017) [34]	weak	moderate	weak	moderate	not app.	**Weak**
Quek (2023) [35]	moderate	strong	moderate	strong	strong	**Strong**
Sanz-Tamargo (2019) [36]	moderate	moderate	moderate	moderate	strong	**Strong**
Tandun (2019) [37]	weak	moderate	weak	strong	strong	**Weak**
**Sum weak**	8	2	9	0	1	***8***
**Sum moderate**	7	8	5	7	2	***2***
**Sum strong**	0	5	1	8	6	***5***
**Sum not applicable**	0	0	0	0	5	***0***
